# Nanomedicine in Lung Cancer Immunotherapy

**DOI:** 10.3389/fbioe.2023.1144653

**Published:** 2023-03-17

**Authors:** Mohammad Doroudian, Saba Zanganeh, Elham Abbasgholinejad, Seamas C. Donnelly

**Affiliations:** ^1^ School of Medicine, Trinity College, Trinity Biomedical Sciences Institute, Dublin, Ireland; ^2^ Department of Cell and Molecular Sciences, Faculty of Biological Sciences, Kharazmi University, Tehran, Iran; ^3^ Department of Clinical Medicine, Trinity College Dublin, Tallaght University Hospital, Dublin, Ireland

**Keywords:** nanomedicine, immunotherapy, lung cancer, nanocarriers, drug delivery

## Abstract

Lung cancer is the major cause of cancer death worldwide. Cancer immunotherapy has been introduced as a promising and effective treatment that can improve the immune system’s ability to eliminate cancer cells and help establish immunological memory. Nanoparticles can contribute to the rapidly evolving field of immunotherapy by simultaneously delivering a variety of immunological agents to the target site and tumor microenvironment. Nano drug delivery systems can precisely target biological pathways and be implemented to reprogram or regulate immune responses. Numerous investigations have been conducted to employ different types of nanoparticles for immunotherapy of lung cancer. Nano-based immunotherapy adds a strong tool to the diverse collection of cancer therapies. This review briefly summarizes the remarkable potential opportunities for nanoparticles in lung cancer immunotherapy and its challenges.

## 1 Introduction

Humankind’s quest to defeat cancer continues by developing targeted treatments. Among the frequently used cancer treatments with significant improvements are chemotherapy, radiation therapy, surgery, and combinations of them. However, these strategies have various limitations; for instance, although surgery offers the best outcome for cancers detected at early stages, this approach often falls short for cancers detected at late stages which have already spread throughout the body. Furthermore, chemotherapy has low specificity, drug-induced side effects, and drug resistance, and has shown higher cancer relapse rates similar to radiation therapy (Velpurisiva et al., 2017; Doroudian et al., 2020; Niloy et al., 2021; Anconina et al., 2022; Hosseinkazemi et al., 2022). As a result, researchers were encouraged to make use of the human body’s own defense system as a tool to fight cancer. Cancer immunotherapy is a rapidly evolving treatment in clinical medicine that enhances the ability of the immune system to recognize and eliminate malignant tumors (Caster et al., 2019).

Cancer immunotherapy has already received FDA approval and can be effective even for late-stage diagnosis. Clinical research has shown that when cancer immunotherapies are effectively formulated into nanoparticles, they generate much stronger and longer-lasting anticancer activity than administered as free drugs, which is an adaptive and enduring treatment method (Chen et al., 2022). Nanoparticles have shown vast improvements in the delivery of immunostimulatory agents through physicochemical manipulation for cancer immunotherapy. In addition to playing a significant role in selective targeting and efficient delivery, material composition and surface modification are also important for stimulating the immune response. Thus, using nanomaterials to engineer cancer immunotherapies is a promising approach worthy of further investigation (Velpurisiva et al., 2017; Doroudian et al., 2019; Farjadian et al., 2022; Hejabi et al., 2022).

As the deadliest cancer, lung cancer has a poor prognosis (Hanjani et al., 2022). Several studies include a broad range of nanoparticles for lung cancer immunotherapy and provide convincing evidence that nanocarriers can stimulate effective immune responses to cancer. An overview of recent advances in lung cancer immune engineering using nanoparticles to improve the therapeutic efficacy of various immunotherapies is presented in this article.

## 2 Immunotherapy

Immunotherapy has brought meaningful improvements in the treatment of patients with cancer. Immunotherapies have limitations because of their immune-related adverse events (irAEs), an immune system, and inflammatory situations against the host’s healthy tissues (Das and Johnson, 2019). The immune system’s action against the host’s tumor is a suitable result, but prediction, diagnosis, and treatment of irAEs is challenging. Immunotherapy has now noticeably introduced itself as one of the most important treatments of cancer, from the metastatic level to the adjuvant and neoadjuvant settings in multiple cancers. Immunotherapy is still at the beginning of improvements, with many obstacles to overcome (Esfahani et al., 2020).

Immune checkpoint inhibitors (ICIs) have demonstrated a significant role in the treatment of non-small-cell lung cancer (NSCLC) patients and have appeared as an effective treatment selection. ICIs have the ability to prevent inhibitory pathways restraining immune response against cancer, restoring, along with sustaining anti-tumor immunity. The blocking agents of 4 Programmed Cell Death Ligand 1 (PD-L1) as well as Programmed Cell Death Protein 1 (PD-1) are adopted in clinics, and using immunotherapy alone or combined with chemotherapy is preferred (Lim et al., 2020). One of the most important members of anti-tumor immunity is T-cell, which plays a critical role in anti-tumor immunity. A great range of immune checkpoint molecules, such as PD-1 (Okazaki and Honjo, 2007), PD-L1 (Herbst et al., 2014), CD73 (Allard et al., 2017), Lag-3 (Andrews et al., 2017), B7-H3 (Suh et al., 2003), TIM-3 (Anderson et al., 2016), CTLA-4 (Krummel and Allison, 1995), TIGIT (Kurtulus et al., 2015), and VISTA (Wang et al., 2011) in the tumor microenvironment (TME) are involved in important mechanisms that prevent effector T cells’ anti-tumor activities. Immune checkpoint antibodies have established meaningful clinical advantages for mono- or combination cancer immunotherapies. The antibody Fc-hinge region binding to Fc gamma receptors is recognized to deeply affect antibody function as well as *in vivo* efficacy. The analysis of immune checkpoint antibodies depends primarily on their effector functions, and its impact on therapeutic efficiency has recently attracted attention (Chen et al., 2019).

**FIGURE 1:**
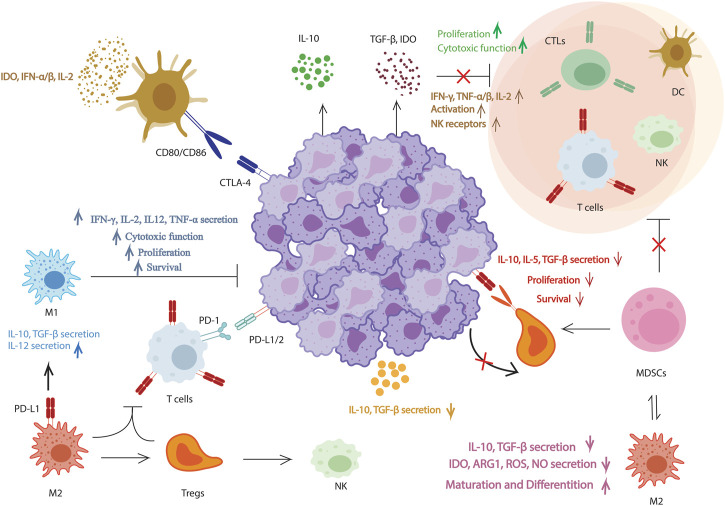
Proposed pathways for molecular procedures of natural immunomodulatory effects. Natural products have inhibitory effects on tumors by enhancing effector T-cells and natural killer cells, reducing CD4+ CD25+ Foxp3+ regulatory T-cells (Tregs), decreasing myeloid-derived suppressor cells, lowering TGF-, IL-5, IL-10, indoleamine 2,3-dioxygenase, and ARG1 levels, promoting IL-12, IFN-γ, and TNF-α/β production, and inhibiting PD-L1 and CTLA-4 in order to prevent Treg cell-cell interaction.

Immunotherapies depending on T cells have achieved remarkable results in cancer treatment, however, assorted challenges exist, such as severe side effects, low response rate, and the acquired resistance, causing unsuitable outcomes. The immunosuppressive TME and the dysfunctional anti-tumor T cells characterize cancer immunity. TME involves tumor cells, vascular elements, stroma, tumor-draining lymph nodes, alongside the biomolecules surrounding them. In TME, numerous cellular and molecular reactions play a key role in shaping the anti-tumor immune mechanism and determining the conditional effectiveness of immunotherapy. In general, T-cell-mediated antitumor immunity involves the following steps in order: In the first step, dying tumor cells produce neoantigens that are caught by antigen-presenting cells (APCs); In the second, APCs show the captured antigens on MHC-I and MHC-II molecules to T cells; The third one is the particular activation of effector T cells against the cancer-specific antigens; In the next level the stimulated effector T cells circulate to tumor site; In the following step T cells enter the tumor; Finally, activated effector T cells bind specifically to cancer cells, and antigen-specific T cells destroy cancer cells (Figure 1) (Deng et al., 2020).

In most cases, immunotherapy does not work or unavoidably develops opposition to treatment after a period, manifested by primary resistance or acquired resistance after the initial response. At the moment, various immunotherapy resistance mechanisms have been recognized, and more keep on being exposed. It is through such efforts that we can improve cancer diagnosis and treatment, and ultimately deliver accurate individualized treatment for patients (Steven et al., 2016; Bai et al., 2020). Immunotherapy based on antibodies has some drawbacks including immunogenicity, membrane permeability, stability, along with production costs. As a result, replacing mechanisms involving small-molecule-regulated immune response are presented (Zhu and Li, 2018).

There are several ongoing immunotherapies for lung cancer including Programmed cell death protein 1 (PD-1) inhibitors, Programmed death-ligand 1 (PD-L1) inhibitors, Chimeric antigen receptor (CAR) T-cell therapy, Oncolytic viruses, and Cancer vaccines. The success rates, failures, and limitations of immunotherapies for lung cancer can vary depending on the specific type of immunotherapy and the individual patient’s circumstances. Pembrolizumab (Herbst et al., 2016) and Nivolumab (Borghaei et al., 2015) have both been shown to improve overall survival in patients with advanced NSCLC. In one study, patients treated with pembrolizumab had a median overall survival of 10.4 months, compared to 6.1 months for those treated with chemotherapy. However, not all patients respond to PD-1 inhibitors, and the response rates can vary widely depending on factors such as the level of PD-L1 expression in the tumor. In one study, the overall response rate to pembrolizumab was 44.8% in patients with PD-L1 expression of 50% or greater, compared to 16.7% in patients with PD-L1 expression of less than 1%. Side effects of PD-1 inhibitors can also occur, with fatigue, nausea, and diarrhea being some of the most common (Borghaei et al., 2015; Herbst et al., 2016). Furthermore, Atezolizumab (Rittmeyer et al., 2017), Durvalumab (Antonia et al., 2017), and Avelumab (Paz-Ares et al., 2018; Borghaei et al., 2020; Paz-Ares et al., 2020) have also been shown to improve overall survival in patients with advanced NSCLC. In one study, patients treated with atezolizumab had a median overall survival of 15.7 months, compared to 11.4 months for those treated with chemotherapy. As with PD-1 inhibitors, response rates to PD-L1 inhibitors can vary depending on factors such as PD-L1 expression levels. In one study, the overall response rate to atezolizumab was 38% in patients with high PD-L1 expression levels, compared to 16% in patients with low PD-L1 expression levels. Side effects of PD-L1 inhibitors can include fatigue, cough, and decreased appetite (Antonia et al., 2017; Rittmeyer et al., 2017; Paz-Ares et al., 2020). Moreover, CAR T-cell therapy is a newer type of immunotherapy that has shown promise in treating certain types of blood cancers. In one study, 7 out of 11 patients with NSCLC who received CAR T-cell therapy had a partial response or stable disease, with a median duration of response of 5.3 months. However, CAR T-cell therapy can also cause serious side effects, such as cytokine release syndrome and neurotoxicity, which occurred in 9 out of the 11 patients in the aforementioned study (Liu B. et al., 2017). There are limited statistics available on the success rates of oncolytic viruses and cancer vaccines in lung cancer, as these types of immunotherapy are still being studied in clinical trials. One phase two trial of a cancer vaccine in patients with NSCLC found that the vaccine improved progression-free survival compared to placebo, but did not improve overall survival (Butts et al., 2005; Butts et al., 2011).

Nanomedicine has shown potential in improving the delivery and efficacy of immunotherapeutic treatments in lung cancer. Nanoparticles can be designed to specifically target cancer cells, thereby reducing toxicity to healthy cells and improving drug delivery to the tumor site. Nanoparticles can be engineered to enhance the immune response against cancer cells and be used to deliver multiple immunotherapeutic agents simultaneously, allowing for combination therapy that can target different pathways involved in cancer progression. Nanomedicine-based immunotherapeutic treatments hold great promise for improving the effectiveness of lung cancer treatments. However, more research is needed to fully understand the potential of this approach and to develop safe and effective nanomedicine-based immunotherapies for clinical use (Chen et al., 2020; Chauhan et al., 2021; Jin et al., 2021; Wang et al., 2022).

## 3 Nanoparticle-based immunotherapy

Nanoparticles with immunomodulatory functions can trigger immune cells and adjust TME to improve antitumor immunity (Yang Z. et al., 2020). Cancer nanomedicine combined with immunotherapies promise an enhancement in the anti-tumor immune responses and sensitization of tumors to immunotherapies (He et al., 2021; Ghasempour et al., 2022). Cancer immune therapeutics limitation is the ability to deliver cancer antigens to immune cells. Nanoparticle systems have a variety of types, among which polymer-based nanoparticles are the most popular platform for cancer immunotherapy and are approved by FDA. A broad range of polymers such as poly (lactide-o-glycolic) acid, chitosan, and polyethylene glycol, have favorable characteristics like biodegradability, biocompatibility, as well as non-toxic features (Buabeid et al., 2020; Doroudian et al., 2021b).

Cancer immunotherapy is limited and cannot respond to treatments because of insufficient efficacy, toxicity concerns, and adverse side effects. Incorporating pH-responsive nanoparticles into immunotherapy brings great access to tackle these disputes since they can target the tumor tissues and organelles of APCs in acidic microenvironment (Yan and Ding, 2020).

The structure and function of TME impinge on the development and metastasis of tumors. TME modulation methods have recently become popular in cancer immunotherapy. Nanoparticles with special environmental characteristics and sophisticated arrangements can efficiently infiltrate TME and significantly deliver the specific components to TME (Yang et al., 2021).

**FIGURE 2:**
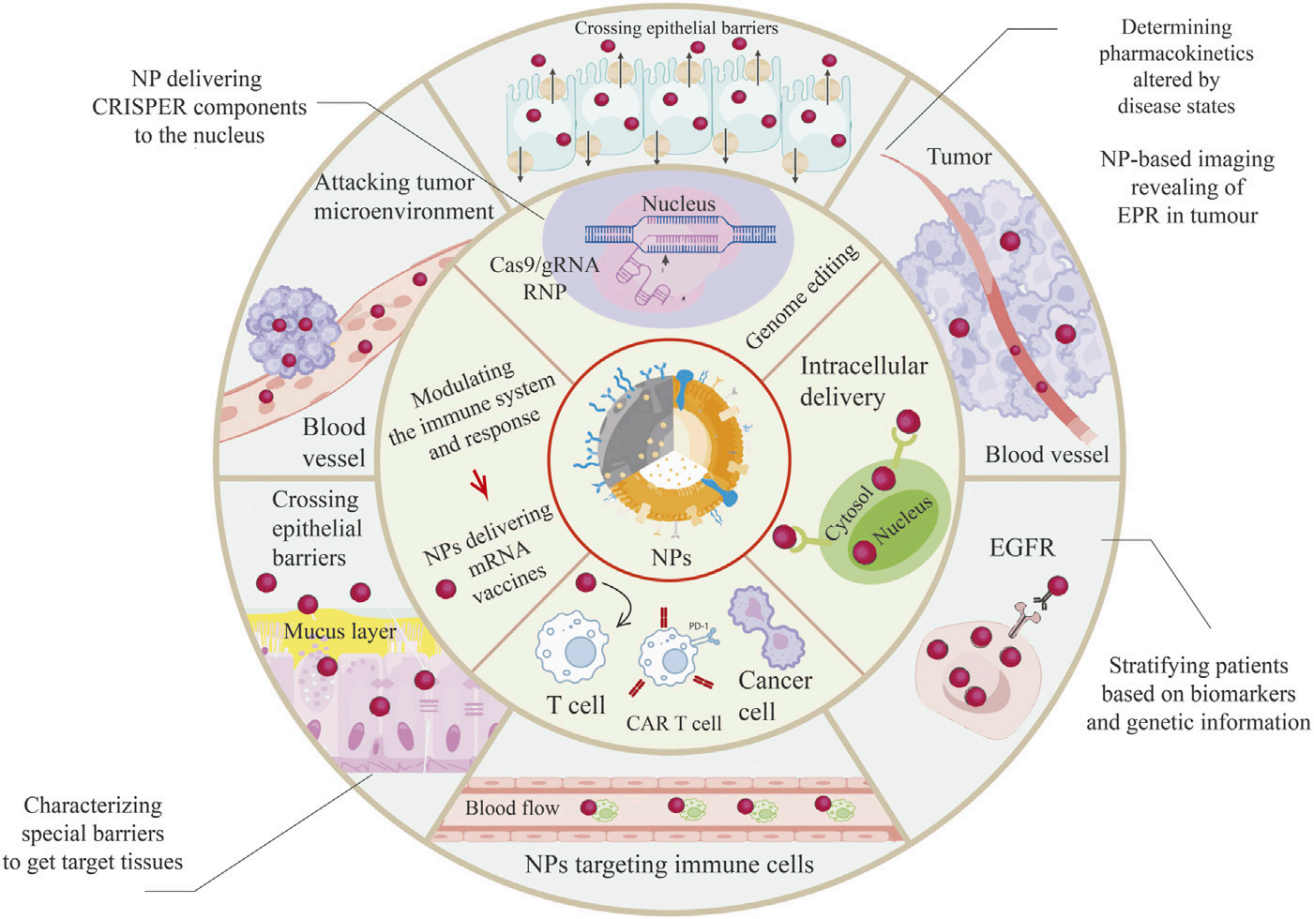
A broad range of applications of Nanoparticles (NPs) in the field of lung cancer treatment. NPs have been increasingly used to improve drug delivery and enhance the effectiveness of precision medicine. NPs can overcome biological barriers such as the blood-brain barrier, which can limit the effectiveness of certain drugs. By encapsulating drugs in NPs, it is possible to improve their solubility, bioavailability, and stability, and target them to specific cells or tissues. For instance, RNA nanoparticles (RNPs) have shown promise in delivering therapeutic RNAs such as siRNA and miRNA for the treatment of various diseases, including cancer and genetic disorders. Similarly, guide RNA (gRNA) delivered *via* NPs has been used in gene editing applications such as CRISPR-Cas9. Enhanced permeation and retention (EPR) is another mechanism that NPs can exploit to improve drug delivery. EPR takes advantage of the leaky blood vessels and poor lymphatic drainage of tumors to accumulate NPs at the tumor site, allowing for targeted drug delivery and minimizing systemic side effects. Epidermal growth factor receptor (EGFR)-targeted NPs have also been used for targeted drug delivery to cancer cells, while chimeric antigen receptor (CAR) T-cell therapy uses NPs to engineer T cells to target and destroy cancer cells. They can be used to deliver CARs directly to T cells, stimulate CAR T cells to proliferate and persist in the body, silence genes that inhibit CAR T-cell function or promote their exhaustion, and deliver CAR T cells directly to the tumor site with enhanced specificity, efficacy, and safety of this promising cancer treatment. Overall, the use of NPs in precision medicine applications holds great promise for improving drug delivery and enhancing the effectiveness of precision medicine therapies.

Nowadays, nanoparticles are implemented in both recognition and treatment. They can be used as adjuvants, able to perform immune-modulating activities, or as a transporter for molecules to be passaged to a special target, after all charged with specific ligands favoring specific uptake (Figure 2) (Di Gioacchino et al., 2020). Cancer immunotherapy is being raised as a significant therapeutic method, and nanoparticles may contribute as an absolute blueprint for systemic delivery of the immune modulators as shown in Table 1 (Castro et al., 2020).

**TABLE 1:** Drug delivery using nanoparticles for lung cancer treatment.

Polymeric nanoparticles	Application	Size	Drug	Main materials	Testing model/cell lines	Method and special features	Ref
**PTX/PCEC nanoparticles**	Antineoplastic agent	168 nm	Paclitaxel	PCL-PEG-PCL, PCEC	A549 cells	A thin film dispersion method. In this work, a passive targeting paclitaxel-loaded nanoparticles (PTX-NPs) was prepared and used to evaluateits synergistic anti-tumor effect by combination with circadian chronomodulated chemotherapy in xenografted human lung cancer	Hu et al. (2017)
**PGSC-PTX NPs PGSC-PTX@RBCm NPs**	Inhibiting tumor growth	Average sizes about 50 nm and 100 nm	Paclitaxel (PTX)	PGSC	NCI-H460 cell line BABL/C nude mice	The emulsification-solvent evaporation method, by using erythrocyte membrane (RBCm) packaging and pH-sensitive technology, pH-sensitive polyethylene nanoparticles (L-γ-glutamyl carbocysteine) -paclitaxel (PGSC-PTX) (PGSC-PTX @ RBCm NPs) was prepared	Gao et al. (2017)
**CSAC and CAP NPs**	Carrier	Ranged from 124 nm to 195 nm and aggre-gated up to 195 nm	5-Fluorouracil (5-FU)	Chondroitinsul-fate tailored cellulose acetate phthalate (CSAC) core shield nanoparticles (NPs)	A549 cancer cell line	Nano- precipitation The CSAC NPs loaded with 5-FU with small particle size were obtained using the nano- precipitation method with a high effect of trapping 5-FU	Garg et al. (2019)
**TiO**_***x***_ **/DOX NPs**	A light activated drug carrier	200 nm	Doxorubicin (DOX)	Titanium peroxide (TiO*x*) nanosheets	A549 cells	Measuring the viabilities of A549 cells investigate the chemotherapy performance, which incubated with free DOX, TiO_2_/DOX and TiO*x*/DOX. For 48 h cells include free DOX and nanocomposites were incubated	Dai et al. (2018)
**SCND-SIS3**	Self-carried nanodrug	280 nm	Smad3 (SIS3)	A self-carried nanodrug-SIS3 (SCND-SIS3)	Lewis Lung Cancer (LLC) mouse model, TGF-β/Smad3-dependent immunoregulatory landscape in NK cells	This NPs based on the reprecipitation method, which largely has an ability to increase solubility and bioavailability while decreases its nephrotoxicity	Lian et al. (2022)
**Abraxane (Nanoparticle Albumin-Bound Paclitaxel)**	Carrier	130 nm	Paclitaxel incorporated	Albumin nanoparticles	Antigen presenting cells (APCs) non-small-cell lung cancer patients (NCT02367781, IMpower130)	The main mechanism of paclitaxel for cancer therapy includes microtubule stabilization. It can block cell division at the G2/M phase	Shi (2020)

## 4 Nanoparticles in lung Cancer immunotherapy

A number of studies have been conducted on nanoparticles as passive delivery routes for cancer therapy, particularly for lung cancer. Cancer cells can be precisely identified using this targeting mechanism by interacting with specific ligands and receptors expressed abundantly in cancer cells (Wathoni et al., 2022). Ongoing and completed clinical trials have already reported positive results using nano-immunotherapy, and preclinical studies have also demonstrated significant improvements in therapeutic efficacy (Sun et al., 2020).

There is evidence that ICI treatment targeting PD-1 and PD-L1 can improve survival in patients with NSCLC. ICIs are only effective in a minority of NSCLC patients, and therefore, superior immunotherapy is promptly required. Reda et al. have designed a nanoparticle-based immunotherapy system called Antigen Release Agent and Checkpoint Inhibitor (ARAC) in order to improve the PD-L1 inhibitor’s efficiency. In the ARAC platform, a nanoparticle co-delivers both Volasertib, the inhibitor of Polo-like kinase 1 (PLK1), along with PD-L1 antibody. In NSCLC and multiple other cancers, PLK1 is overexpressed as a key mitotic kinase that stimulates cell proliferation. Through the inhibition of PLK1, cancer cells are selectively killed, and PD-L1 expression is upregulated in surviving cancer cells, resulting a feedforward targeted delivery of ARAC using PD-L1 antibody-conjugated nanoparticles. It reduces volasertib and PD-L1 antibody effective doses by five times in a metastatic lung tumor model (LLC-JSP) by primarily influencing CD8^+^ T cells. Furthermore, ARAC has been shown to be effective in KLN-205, a lung tumor model that is not responsive to the combinations of CTLA-4 and PD-1 inhibitors (Liu Z. et al., 2017; Zhang et al., 2020; Islam and Stanslas, 2021; Reda et al., 2022).

It has been proposed that blocking the PD-1/PD-L1 pathway would be a promising immunotherapeutic approach, but the response rate of their inhibition is low, resulting in minimal infiltration of cytotoxic T cells. Using an immunosuppressive microenvironment to alleviate intractable tumor microenvironments, Wang et al. developed a novel mesoporous nanocarrier with a core made of upconverting nanoparticles and a shell made of large pores of mesoporous silica (UCMS) simultaneously loaded with photosensitizer molecules, the AL-9 peptide vaccine from the IDO, and the inhibitors of PD-L1, and tested it in Lewis murine lung carcinoma cell line and mouse model. The components of the designed UCMS@Pep-aPDL1 were shown to be capable of potentiated local and systemic anti-tumor immunity through stimulating the killing of IDO-expressed tumor cells, inducing immunogenic cell death, and promoting the infiltration of effector T-cell (Wang et al., 2021; Chen et al., 2022).

The STK11 gene (LKB1), encoding serine/threonine kinase 11, is deleted or inactivated in nearly half of NSCLC patients with activated KRAS mutations. KRAS mutant NSCLC metastatic tumors are mostly resistant to anti-PD-L1 or PD-1 immunotherapy in nature. An animal model of humanized mice has been used by Meraz et al. to indicate that whereas carboplatin and pembrolizumab inhibit tumor growth moderately and transiently, TUSC2, a tumor suppressor gene delivered in nanovesicles, eradicates tumor growth in most animals. A pro-immune microenvironment in the tumor is modulated by TUSC2, which makes KRAS/LKB1 tumors more sensitive to carboplatin plus pembrolizumab. They also found that TUSC2 restoration reduced PD-L1 expression and synergized with anti-PD-1 treatment in KRAS mutant lung cancer models of mice (Meraz et al., 2018; Meraz et al., 2022).

Toll-like receptor (TLR) agonists are immunomodulators capable of inducing innate immune responses and controlling adaptive immunity which have attracted attention as anticancer molecules in the early stages of clinical development. CpG oligodeoxynucleotides bind to TLR9 as a TLR9 agonist and evoke a cascade of innate and adaptive immune responses. CpG stimulates dendritic cells and causes secondary effects such as pro-inflammatory cytokines secretion, natural killer cell activation, and T-cell expansion as well as superior antitumor effects while being locally delivered in tumors. Perry et al. (2020) have employed Particle Replication In Non-wetting Templates (PRINT) nanoparticles for the delivery of CpG into two murine orthotopic metastasis lung cancers (NSCLC-344SQ and KAL-LN2E1) *via* orotracheal instillations. The PRINT-CpG Nano-platform local delivery into lungs resulted in extensive tumor regression, resistance to tumor rechallenge, sustained boost of antitumor cytokine levels and extended CpG residence time in the lungs, as well as lower toxicity compared to soluble CpG .

MicroRNAs (miR) are small endogenous non-coding RNAs, which are actively involved in the adjustment of adaptive and innate immune responses. Some miRs including miR-155, miR-125, as well as miR-29 are able to stimulate repolarization of tumor-associated macrophages (TAMs) from pro-tumor M2 phenotype towards antitumor/pro-inflammatory M1 phenotype. Macrophage activation is regulated by miR-125b, which is expressed at a higher level in macrophages compared to other immune cells. By promoting the M1 macrophage phenotype through miR-125b overexpression by a virus-based vector, the anti-tumor efficacy is enhanced by improving the killing of cancer cells directly or inhibiting their proliferation in an indirect manner in a model of subcutaneous EL4 tumor. By using hyaluronic acid-poly (ethylenimine) (HA-PEI)-based nanoparticles with encapsulated miR-125b to target CD44, Parayath et al. (2018) demonstrated macrophage-specific delivery and transfection after intraperitoneal administration. The miR-125b transfectants increased the ratio of M1 to M2 macrophages by > 6 fold as well as iNOS (M1 marker) and Arg-1 (M2 marker) ratios in TAMs by 300 fold over the untreated controls, indicating the successful repolarization of TAMs in a KRAS/p53 double mutant genetically engineered NSCLC mouse model and naïve mice through the intraperitoneal administration of macrophage-specific HA-PEI nanoparticles in their lung tissues as a promising strategy in anticancer immunotherapy (Gharavi et al., 2022; Zanganeh et al., 2022).

Other viral vectors have also been used as a nanotechnology tool for immunotherapy against cancer. Inhaling self-assembled virus-like nanoparticles from Cowpea Mosaic Virus, as an immunostimulatory agent, triggers neutrophils in the microenvironment of tumors and interacts with the immune system, which has been studied by Lizotte et al. in lung cancer metastasis. Furthermore, Feldmann et al. (2018) designed an adenovirus-inspired polymer system for endosomal release and linked this nanoparticle to siRNA, delivered it to NCI-H1299 cells which is a NSCLC cell line in humans, and knocked down the genes involved in cancer development or progression (Lizotte et al., 2016; Carrasco-Esteban et al., 2021).

Combination therapy has also shown promising potential for cancer treatment. Yang et al. have developed hyaluronic acid-cisplatin/polystyrene-polymetformin dual-prodrug co-assembled nanoparticles as a combination therapy against lung cancer which can overcome the asynchronous pharmacokinetic behavior barriers of cisplatin and metformin occurring while utilized in the free drug form. The results of *in vitro* and *in vivo* tests of this nano-platform on Lewis lung cancer cells include higher tumor accumulation and proliferation inhibition, induction of apoptosis, increase in CD4^+^ and CD8^+^ T cells as well as the expression of TNF-α and IFN-γ cytokines, and prolonged overall survival of mouse models through this potent immunotherapeutic function (Yang T. et al., 2020).

Chiang et al. have designed a novel drug delivery platform including magnetic Iron Oxide nanoparticles along with Fucoidan accompanied by aldehyde-functionalized dextran (Dex) and combined it with T-cell activators and checkpoint inhibitors. As the ICI, they added anti-PD-L1 antibody and supplemented it with anti-CD3/CD28 antibody to act as the activator of the immune system. The application of IO-FuDex3-nano-biohybrid immunotherapy in a 4T1-metastatic lung carcinoma model resulted in low systemic toxicity, higher infiltration CD4^+^ and CD8^+^ T cells, lower number of metastatic nodules, improved immune responses, and an extended median survival rate of animals (Chiang et al., 2018; Rawal and Patel, 2021; Sanaei et al., 2021).

Su et al. demonstrated the potential of Au@PG nanoparticles consisting of Au and polyaniline-based glyco structures. Employing Au@PG nanoparticles in lung cancer immunotherapy resulted in the promising polarization activity of M1 macrophage and the promotion of the cytotoxic T cell response as well as tumor inhibition and the secretion of immunogenic cytokines when combined with PD-1 therapy (Su et al., 2022). Another active targeted nanoparticle is SGT-53 which is capable of restoring the function of the p53 protein and improving anti-PD-1 immunotherapy by delivering the TP53 tumor suppressor gene to tumor cells. SGT-53 has gone through several stages of clinical trials and is considered a potent strategy against lung cancer (Kim et al., 2018; Doroudian et al., 2021a).

There are some specific limitations in immunotherapy for lung cancer compared to other cancers where nanomedicine can aid in developing better treatments including limited response rates, toxicity, and resistance. Immunotherapy has shown limited response rates in lung cancer compared to other cancer types, such as melanoma and renal cell carcinoma. This may be due to the complex immune environment of lung cancer, which involves multiple immune suppressive pathways (Gettinger et al., 2018; Hellmann et al., 2018). Furthermore, immunotherapy can cause significant toxicity, particularly when used in combination with other treatments such as chemotherapy. This can limit the dose and duration of treatment, which can impact treatment efficacy (Gandhi et al., 2018; Postow et al., 2018). Some lung cancers may also develop resistance to immunotherapy, leading to treatment failure. This may be due to multiple factors, including the development of new mutations, alterations in the tumor microenvironment, and immune escape mechanisms (Pardoll, 2012; Dong et al., 2017; Skoulidis et al., 2018). Nanomedicine has the potential to address some of these limitations by improving the delivery and efficacy of immunotherapeutic treatments in lung cancer. For example, nanoparticles can be designed to specifically target cancer cells and reduce toxicity to healthy cells, potentially improving treatment response rates and reducing toxicity. Additionally, nanoparticles can be used to deliver multiple immunotherapeutic agents simultaneously, allowing for combination therapy that can target different pathways involved in cancer progression (Shi et al., 2017; Cheng et al., 2021; Jagdale Swati et al., 2023).

As advanced drug delivery vehicles, nanoparticles are expected to leverage existing platforms to enhance specific subpopulations’ targeting as well as co-stimulatory signals’ synchronous delivery. Nanomedicines with novel properties, including engineered immune mimics, may have the greatest potential to alter paradigms in immunotherapy.

## 5 Conclusion and perspectives

Nanoparticle immunotherapy is demonstrating tremendous potential at its early stages of development. Nanoparticles can deliver the optimal dose of immunotherapy to the target region without stimulating adverse side effects. These versatile nano-platforms can be engineered in a variety of characteristics such as shape, size, elasticity, kinetics, surface modifications, and specific targeting. Smart nanoparticles can be formulated with stimuli-sensitive materials to limit immunotherapeutic release to when the engineered nanoparticles arrive at their target tumor microenvironment and specific destination. Many factors must be considered and enhanced before nanoparticles can be clinically adopted for the immunotherapy of lung cancer. The design of nanoparticle immunotherapies should be improved for a better targeting specificity. The efficacy of nanoparticle immunotherapies which are under development stages should be boosted so that their application over conventional therapies would be justified. Mechanistic, *in silico*, and *in vivo* studies need to be conducted to determine the immune responses to this therapy and how it evolves over time, the homogenous size biodistribution of nanoparticles, the toxicity and safety concerns of such approach, and the immunogenicity of these nano-delivery vehicles as well as the therapeutics they carry. Additionally, a comprehensive grasp of the tumor biology, its microenvironment, and all the interactions between cancer cells and nanoparticles should be obtained for nanoparticles to be able to be developed for immunotherapy of lung cancer tumors and its metastases.
